# Comparison of complications and oncology outcomes of immunotherapy alone and combination in metastatic castration-resistant prostate cancer (mCRPC): a systematic review and meta-analysis

**DOI:** 10.1097/JS9.0000000000003309

**Published:** 2025-08-27

**Authors:** Jun Liu, Yong-Ming Kang, You-Gang Feng, Cai Zhang, Wen-Zhi Chen

**Affiliations:** a Department of Urology, Suining Central Hospital, Suining, China; b State Key Laboratory of Ultrasound in Medicine and Engineering, College of Biomedical Engineering, Chongqing Medical University, Chongqing, China

**Keywords:** immunotherapy, meta-analysis, metastatic castration-resistant prostate cancer

## Abstract

**Objective:**

Comparison of complications and oncology outcomes of immunotherapy alone and combination in metastatic castration-resistant prostate cancer (mCRPC).

**Methods:**

As of March 2025, a systematic search on the application of immunotherapy alone with immunotherapy combined with other treatments (immunotherapy combined with radiotherapy/endocrine therapy, etc.) in the Embase, PubMed, and Web of Science databases for mCRPC. The complication and oncology outcome data from the study were collected for subsequent analysis. Stata17 software is used for data analysis.

**Results:**

A total of nine studies were included. Compared with immunotherapy alone, immunotherapy combined with other treatments increased the overall survival of patients (effect = 0.67, 95% Cl [0.35, 0.98], *P* < 0.05) and median survival time (effect = 0.74, 95% Cl [0.45, 1.02], *P* < 0.05). The response rate of PSA50 in patients was increased (OR = 3.78, 95% Cl [1.12, 10.77], *P* < 0.05). There was no significant difference in the overall complication rates between the two.

**Conclusion:**

Preliminary findings so far show that immunotherapy in combination with other therapies improves overall survival, median survival time, and PSA50 response rate, with no significant difference in overall complication rate between the two groups. This conclusion is based on the pooled results of nine studies, which are small in number, and more multicenter, high-quality studies are needed to confirm this.

## Introduction

Prostate cancer (PCa) is the leading cause of cancer-related death in men, and due to the lack of effective treatment, most PCa patients receiving androgen deprivation treatment progress to metastatic castration-resistant prostate cancer (mCRPC)^[1]^. However, prostate cancer is a hormone-sensitive tumor. Once converted to mCRPC, it is difficult to inhibit tumor growth and metastasis. Despite the use of chemotherapy and novel androgen receptor signaling inhibitors, mCRPC remains a fatal disease with poor clinical outcomes^[2]^. Tumor immunotherapy blocking checkpoint inhibitors has completely changed the therapeutic prospects for several malignant tumors, but mCRPC has not benefited due to immunosuppression and poor immunogenic “cold” tumor microenvironment (TME), and new therapeutic approaches and targets are needed to be studied^[3,4]^. Sipuleucel-T (sip-T) is the only FDA-approved autologous cell immunotherapy for mCRPC^[5]^. Sipuleucel-T, as the first cancer vaccine for the treatment of asymptomatic or mild symptomatic mCRPC, has ushered in a new era of immunotherapy^[6]^. However, studies have pointed out that the current single immunotherapy has limited benefits for mCRPC. New combinations may enhance the effectiveness of immunotherapy^[7]^.

**HIGHLIGHTS**

- Our study compensates for some of the efficacy of immunotherapy alone and in combination with immunotherapy for castration-resistant prostate cancer.
- We performed a very detailed analysis comparing oncology and complication outcomes between the two groups.
- Immunotherapy combined with other treatments increased the overall survival, median survival time, and PSA50 response rate of patients, and there was no significant difference in the overall complication rate between the two groups.

As early as 2013, a study combined immune checkpoint inhibitors (ICI) with radiotherapy and found that combined treatment of PSA50 was significantly increased compared with immune checkpoint inhibitor treatment alone without additional adverse effects^[8]^. A recent study that combined Sipuleucel-T with radiotherapy also found that combined treatment of PSA50 has significantly increased, with a longer overall survival time and disease-free time to progress^[9]^. These studies seem to suggest that immunotherapy combined with other treatments can lead to better clinical outcomes for patients than immunotherapy alone. Unfortunately, the current topic lacks meta-analysis results with high ratings of evidence. Therefore, our aim in conducting this study according to the TITAN guideline 2025 was to hopefully provide a short guide for clinical practice^[10]^.

## Methods

### Search strategy

This systematic review and meta-analysis was conducted strictly by the PRISMA standard^[11]^ and the AMSTAR^[12]^ standards. Two researchers independently completed the literature search and screening process. The Embase, PubMed, and Web of Science databases were retrieved. The search time is March 2025. The search keywords are as follows: immunotherapy, Sipuleucel-T, ICI, and metastatic castration-resistant prostate cancer. Manually searching related research references to expand the search scope.

### Eligibility criteria

This meta-analysis mainly included nine studies. Inclusion criteria: (1) patients with mCRPC with clear diagnosis, (2) The same study included comparisons of immunotherapy alone (ICI and SIP-T) with immunotherapy combine (ICI/SIP-T combined radiotherapy/endocrine therapy, etc.), (3) at least one outcome indicator of interest, including overall survival (OS), median survival time (MST), progression-free survival (PFS), PSA50, complications, and (4) publication language in English.

Exclusion criteria: (1) do not include comparisons between immunotherapy alone and immunotherapy combine, (2) non-English studies, and (3) studies with incomplete data or lack of critical data.

### Data extraction

The two researchers independently extracted the relevant data of the study, and the final extracted data included: author, year, country, type, race, study period, sample, center, therapy, and outcome indicators of interest, including overall survival (OS), median survival time (MST), progression-free survival (PFS), PSA50, complications.

### Quality assessment

The quality assessment of cohort studies were followed by the Newcastle-Ottawa Scale (NOS)^[13]^, which had a total of nine points, with a score of 0–3 for low-quality studies, a score of 4–6 for medium-quality studies, and a score of 7–9 for high-quality studies. The quality evaluation system of the randomized controlled studies was used to evaluate the quality of the randomized controlled studies.

### Data synthesis

Cochrane Q statistics evaluated inter-study heterogeneity and calculated I^2^ statistics, such as I^2^ > 50% or *P* < 0.10, indicating that inter-study heterogeneity is significant, and the random effect model was selected. Statistical analysis was performed using Stata17. This analysis is based on the “dbmetan” command. This command is usually used to combine ratios and mean differences. For results with high heterogeneity, we used sensitivity analysis to determine whether its heterogeneity has a stable source.

## Results

### Characteristics of the included studies

**Figure 1.**
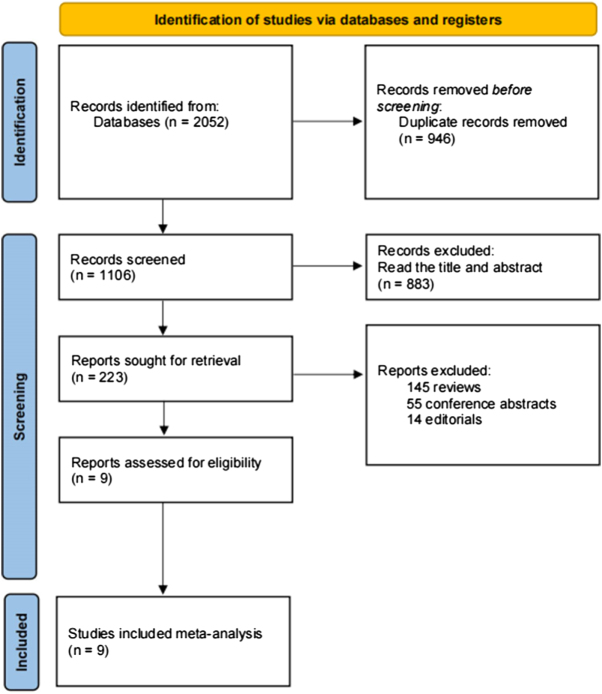
Flow diagram of the study’s selection process.

The author retrieved 2052 records from 3 databases. The 946 duplicate documents were excluded, 883 documents were excluded after reading the title and abstract, 223 documents were included in the careful reading, 145 reviews, 55 conference abstracts, and 14 editorials were excluded. A total of nine studies were included in the meta-analysis^[8,9,14–20]^. Most were cohort studies (Fig. 1).

**Figure 2.**
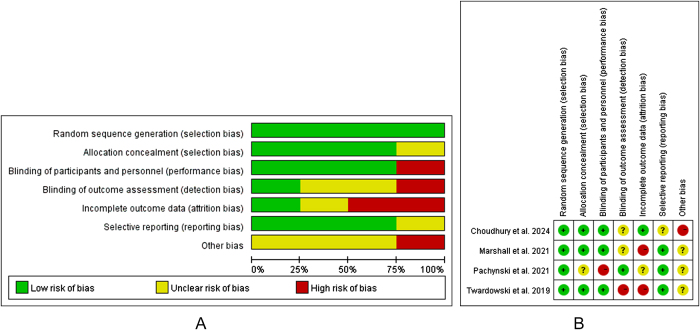
Forest plot and meta-analysis of the quality assessment.

Baseline data for each study (Table 1) include author, year, country, type, race, study period, sample, center, and therapy.

**Table 1:** Baseline data for studies included in the meta-analysis

Author	Year	Country	Type	Race	Study period	Sample (n)	Center	Therapy
Slovin *et al*	2013	USA	Retrospective	Asian, Black, White	2006–2009	75	Multiple center	ICI^b^ alone
								ICI+^c^
Pachynski *et al*	2021	USA	RCT^a^	Black, White	2015–2017	54	Single center	Sipuleucel-T alone
								Sipuleucel-T+
Sinha *et al*	2021	USA	Retrospective	Asian, Black, White	2014–2020	50	Multiple center	ICI alone
								ICI+
Shenderov *et al*	2021	USA	Retrospective	Black, White, other	2016–2020	30	Single center	ICI alone
								ICI+
Aggarwal *et al*	2023	USA	Retrospective	Asian, Black, White	2019–2022	43	Single center	ICI alone
								ICI+
Choudhury *et al*	2024	USA	RCT	NA	2017–2019	45	Single center	ICI alone
								ICI+
Marshall *et al*	2021	USA	RCT	Black, White, other	2017–2018	32	Single center	Sipuleucel-T alone
								Sipuleucel-T+
Ellen *et al*	2018	USA	Two-arm pilot trial	White/Caucasian	2013–2016	17	Single center	Sipuleucel-T alone
								Sipuleucel-T+
Twardowski *et al*	2019	USA	RCT	NA	2014–2020	51	Single center	Sipuleucel-T alone
								Sipuleucel-T+

RCT^a^ = randomized controlled trial; ICI^b^ = immune checkpoint inhibitors; +^c^ = combine other therapy.

### Quality assessment

The included cohort studies were scored using the NOS^[13]^ study quality assessment scale. A total of five studies were included. All are of medium mass (Table 2). The quality evaluation system of the randomized controlled studies was used to evaluate the quality of the randomized controlled studies, and the results are shown in (Fig. 2).

**Table 2:** Quality score of included studies based on the NOS scale

Study	Selection			Comparability			Exposure			Total stars
	aREC	bSNEC	cAE	dDO	eSC	fAF	gAO	hFU	iAFU	
Slovin *et al*	1	1	1	1	1	1				6
Sinha *et al*	1	1	1	1	1		1			6
Shenderov *et al*	1	1	1	1	1	1		1		7
Aggarwal *et al*	1	1	1	1	1	1		1	1	8
Ellen *et al*	1	1	1	1	1				1	7

^a^ REC, representativeness of the cohort;

^b^ SNEC, selection of the non-exposed cohort;

^c^ AE, ascertainment of exposure;

^d^ DO, demonstration that outcome of interest was not present at start of study;

^e^ SC, study controls most important factors;

^f^ AF, study controls for other important factors;

^g^ AO, assessment of outcome;

^h^ FU, follow-up long enough for outcomes to occur;

^i^ AFU, adequacy of follow-up of cohort (≥ 80%).

## Results

### Oncology outcomes

#### Overall survival (OS)

Four studies reported ICI combine versus ICI. Low heterogeneity among studies (I^2^ = 0.0%, *P* > 0.1). The results show that ICI combine’s OS is long (effect = 0.67, 95% Cl [0.08, 1.26], *P* < 0.05).

**Figure 3.**
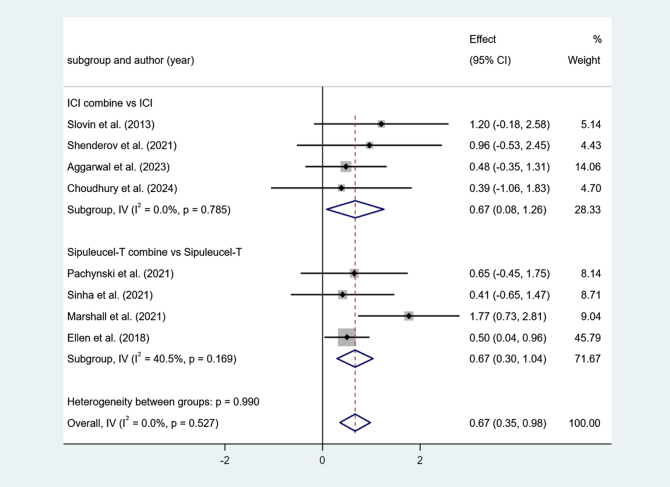
Forest plot and meta-analysis of the OS between immunotherapy alone and combination.

Four studies reported Sipuleucel-T combine versus Sipuleucel-T. Low heterogeneity among studies (I^2^ = 40.5%, *P* > 0.1). The results show that Sipuleucel-T combine’s OS is long (effect = 0.67, 95% Cl [0.30, 1.04], *P* < 0.05) (Fig. 3).

#### Median survival time (MST)

Four studies reported ICI combine versus ICI. Low heterogeneity among studies (I^2^ = 0.0%, *P* > 0.1). The results show that ICI combine’s MST is long (effect = 0.51, 95% Cl [0.01, 1.02], *P* < 0.05).

**Figure 4.**
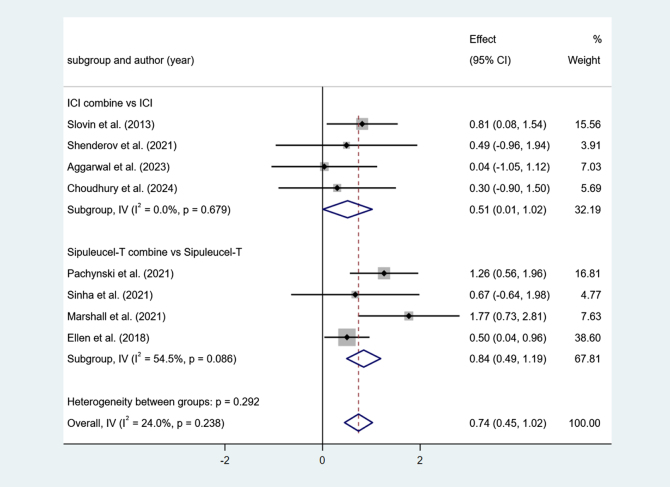
Forest plot and meta-analysis of the MST between immunotherapy alone and combination.

Four studies reported Sipuleucel-T combine versus Sipuleucel-T. Higher heterogeneity among studies (I^2^ = 54.5%, *P* < 0.1). The results show that Sipuleucel-T combine’s MST is long (effect = 0.84, 95% Cl [0.49, 1.19], *P* < 0.05) (Fig. 4).

#### Progression-free survival (PFS)

Two studies reported ICI combine versus ICI. Low heterogeneity among studies (I^2^ = 8.5%, *P* > 0.1). The results show no difference (effect = 0.41, 95% Cl [−0.12, 0.93], *P* > 0.05).

**Figure 5.**
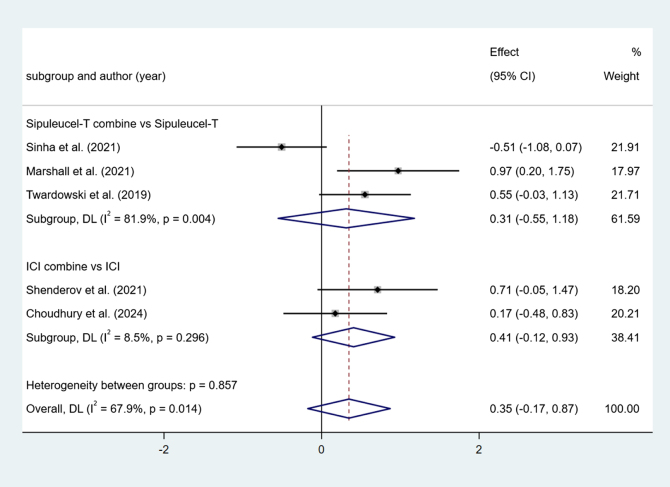
Forest plot and meta-analysis of the PFS between immunotherapy alone and combination.

Three studies reported Sipuleucel-T combine versus Sipuleucel-T. Higher heterogeneity among studies (I^2^ = 81.9%, *P* < 0.1). The results show no difference (effect = 0.31, 95% Cl [−0.55, 1.18], *P* > 0.05) (Fig. 5).

#### PSA50

Four studies reported ICI combine versus ICI. Higher heterogeneity among studies (I^2^ = 53.6%, *P* < 0.1). The results show that ICI combine’s PSA50 decline rate is high (OR = 3.96, 95% Cl [1.70, 9.22], *P* < 0.05).

**Figure 6.**
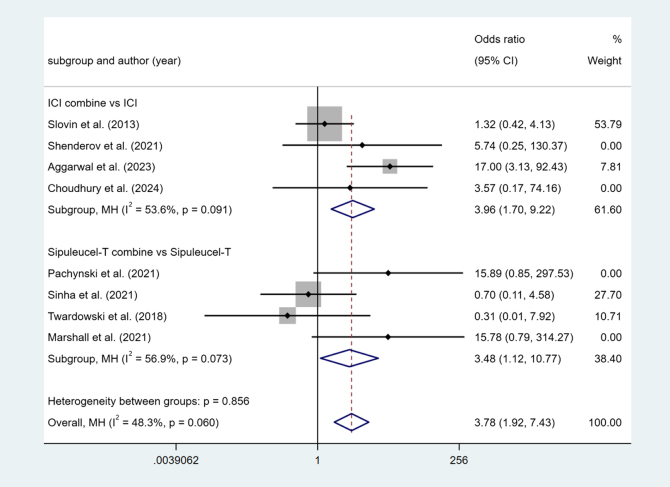
Forest plot and meta-analysis of the PSA50 between immunotherapy alone and combination.

Four studies reported Sipuleucel-T combine versus Sipuleucel-T. Higher heterogeneity among studies (I^2^ = 56.9%, *P* < 0.1). The results show that Sipuleucel-T combine’s PSA50 decline rate is High (OR = 3.48, 95% Cl [1.12, 10.77], *P* < 0.05) (Fig. 6).

### Adverse

#### Overall Adverse (OA)

Four studies reported ICI combine versus ICI. Low heterogeneity among studies (I^2^ = 0.0%, *P* > 0.1). The results show no difference (OR = 1.07, 95% Cl [0.36, 3.21], *P* > 0.05).

**Figure 7.**
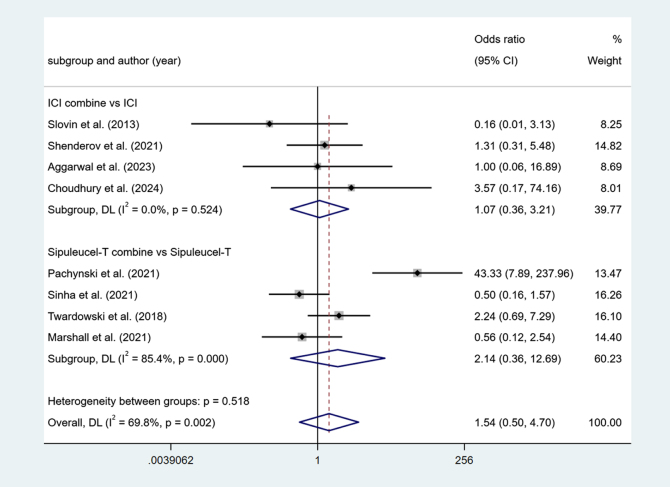
Forest plot and meta-analysis of the OA between immunotherapy alone and combination.

Four studies reported Sipuleucel-T combine versus Sipuleucel-T. Higher heterogeneity among studies (I^2^ = 85.4%, *P* < 0.1). The results show no difference (OR = 2.14, 95% Cl [0.36, 12.69], *P* > 0.05) (Fig. 7).

#### Adverse Grading (AG)

**Figure 8.**
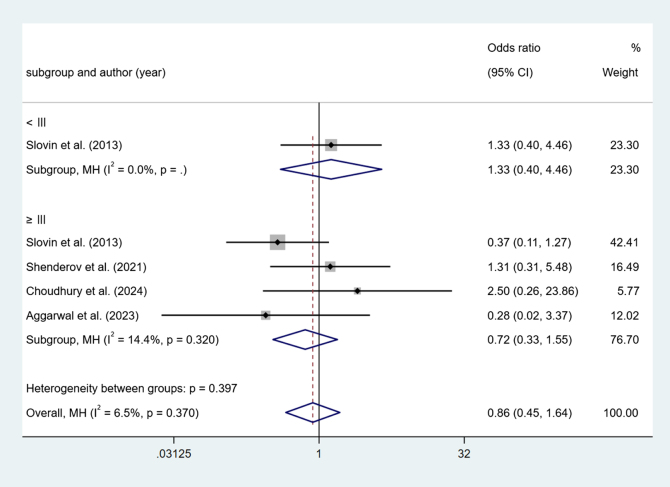
Forest plot and meta-analysis of the AG between ICI combine versus ICI.

In ICI combine versus ICI, 1 study reported AG<III. The results show no difference (OR = 1.33, 95% Cl [0.40, 4.46], *P* > 0.05). Four studies reported AG ≥ III. The results show no difference (OR = 0.72, 95% Cl [0.33, 1.55], *P* > 0.05) (Fig. 8).

**Figure 9.**
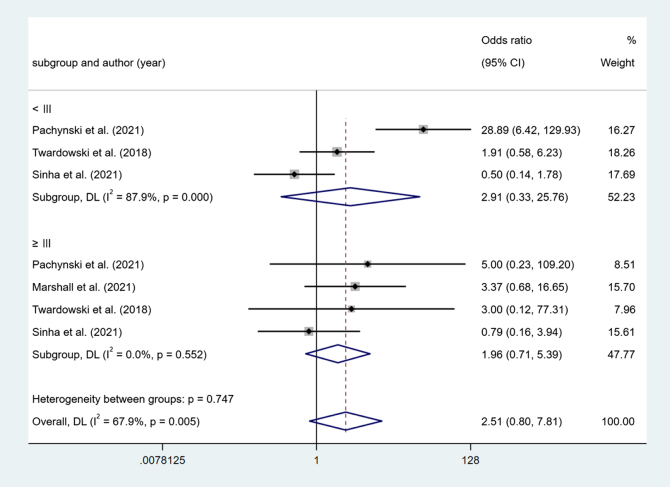
Forest plot and meta-analysis of the AG between Sipuleucel-T combine versus Sipuleucel-T.

In Sipuleucel-T combine versus Sipuleucel-T, 3 study reported AG<III. The results show no difference (OR = 2.91, 95% Cl [0.33, 25.76], *P* > 0.05). Four studies reported AG ≥ III. The results show no difference (OR = 1.96, 95% Cl [0.71, 5.39], *P* > 0.05) (Fig. 9).

#### Fatigue

Four studies reported ICI combine versus ICI. Higher heterogeneity among studies (I^2^ = 75.6%, *P* < 0.1). The results show no difference (OR = 1.54, 95% Cl [0.36, 6.63], *P* > 0.05).

**Figure 10.**
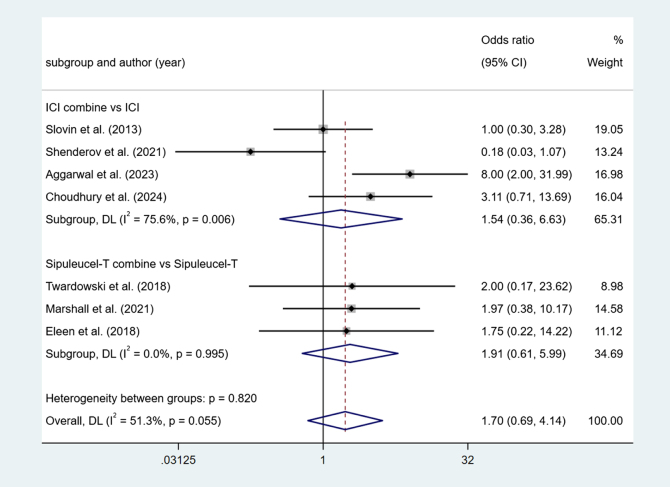
Forest plot and meta-analysis of the Fatigue between immunotherapy alone and combination.

Three studies reported Sipuleucel-T combine versus Sipuleucel-T. Low heterogeneity among studies (I^2^ = 0.0%, *P* > 0.1). The results show no difference (OR = 1.91, 95% Cl [0.61, 5.99], *P* > 0.05) (Fig. 10).

#### Pain

Two studies reported ICI combine versus ICI. Higher heterogeneity among studies (I^2^ = 79.6%, *P* < 0.1). The results show no difference (OR = 0.10, 95% Cl [0.00, 2.17], *P* > 0.05).

**Figure 11.**
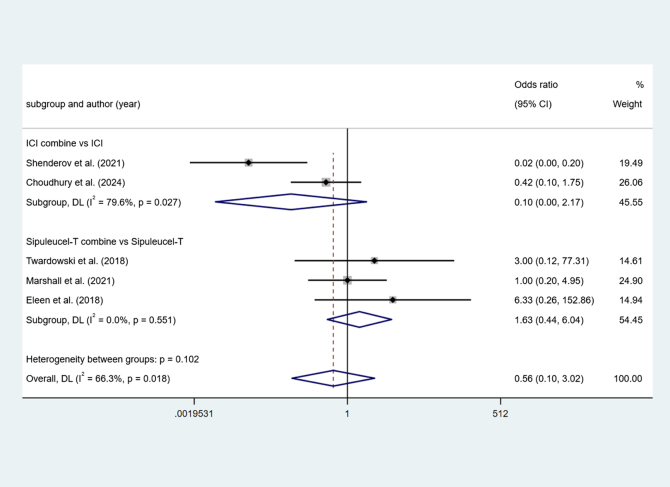
Forest plot and meta-analysis of the Pain between immunotherapy alone and combination.

Three studies reported Sipuleucel-T combine versus Sipuleucel-T. Low heterogeneity among studies (I^2^ = 0.0%, *P* > 0.1). The results show no difference (OR = 1.63, 95% Cl [0.44, 6.04], *P* > 0.05) (Fig. 11).

#### Anemia

Four studies reported ICI combine versus ICI. Low heterogeneity among studies (I^2^ = 40.3%, *P* > 0.1). The results show no difference (OR = 1.20, 95% Cl [0.52, 2.77], *P* > 0.05).

**Figure 12.**
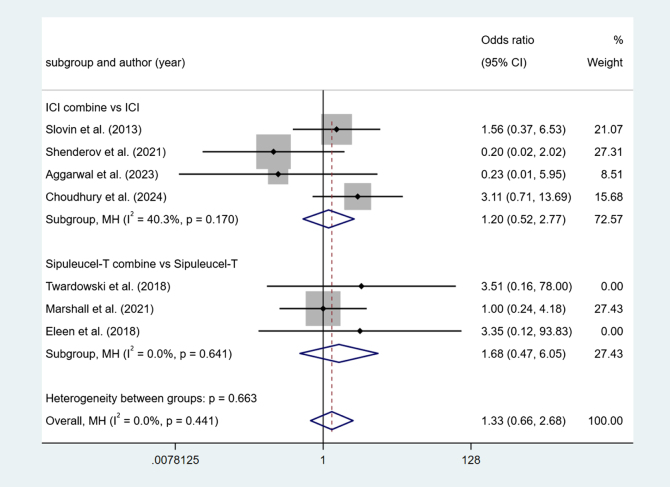
Forest plot and meta-analysis of the Anemia between immunotherapy alone and combination.

Three studies reported Sipuleucel-T combine versus Sipuleucel-T. Low heterogeneity among studies (I^2^ = 0.0%, *P* > 0.1). The results show no difference (OR = 1.68, 95% Cl [0.47, 6.05], *P* > 0.05) (Fig. 12).

## Sensitivity analysis

We conducted sensitivity analysis to determine whether studies with higher heterogeneity have stable sources of heterogeneity. This enhances the reliability of our research results. The results showed that PFS, pain, and overall adverse had stable heterogeneous sources.

## Discussion

Metastatic castration-resistant prostate cancer (mCRPC) is a disease with an extremely poor prognosis and has limited efficacy in traditional treatments^[21]^. In recent years, the rise of immunotherapy has brought new hope for mCRPC treatment, but the efficacy of immunotherapy alone is not ideal^[22]^. Therefore, exploring the model of combined immunotherapy has become a hot topic in current research. Through meta-analysis, this study comprehensively evaluated the adverse reactions and oncological results of immunotherapy alone and immunotherapy combined with other treatments in mCRPC, aiming to provide a reference for clinical practice.

From the perspective of adverse reactions, immunotherapy alone (such as sipuleucel-T or pembrolizumab) is usually well tolerated, but the incidence of immune-related adverse events (irAEs) cannot be ignored. Adverse of immunotherapy alone are mainly concentrated in the skin, gastrointestinal tract, endocrine systems, and can usually be managed through standard treatment options^[23]^. For example, in the study of sipuleucel-T, adverse events such as itching, fatigue, chills and fever were more common, but most were grade 1-2 and had fewer serious adverse events^[14,17]^. However, when immunotherapy combined with other therapies, such as radiotherapy, the incidence and severity of adverse events may increase. For example, in studies combined with radiotherapy, the incidence of grade 3/4 adverse events is higher, including diarrhea, colitis, and hepatitis^[16]^. Chemotherapy combined with immunotherapy may increase hematologic toxicity and risk of infection^[24]^. In addition, combination therapy may also trigger some unique adverse events, such as in the study of sipuleucel-T combined with radiotherapy, where some patients experienced transient lymphopenia^[19]^. This suggests that when choosing a combination therapy regimen, we need to weigh the risk of treatment effects versus adverse events. Interestingly, our meta-analysis results show that there is no significant difference between immunotherapy alone and combined treatment in terms of overall adverse, and common adverse reactions such as fatigue, pain, and anemia. It is worth noting that our results were derived from 9 studies, which is a more restricted number of studies, and that we compared immunotherapy alone with combination immunotherapy. There was no split of combination immunotherapy into specific regimens, such as immunotherapy combined with radiotherapy, endocrine therapy, and other specific directions. This may lead to greater heterogeneity in our results, with some errors that mask the actual evaluation of adverse effects occurring in different combinations. Therefore, our results are only a preliminary result and further validation is needed subsequently. Please be cautious in citing our results when it comes to the therapeutic regimen of a specific combination.

In terms of oncological outcomes, the efficacy of immunotherapy alone is relatively limited. For example, pembrolizumab monotherapy has an objective response rate (ORR) of only about 5% in mCRPC, and most patients cannot benefit from treatment^[14]^. In contrast, combined immunotherapy has shown certain efficacy advantages. For example, in the study of sipuleucel-T combined with IL-17, some patients experienced a significant decrease in PSA levels and the treatment response lasted for a long time^[16]^. In addition, studies of radiotherapy combined with sipuleucel-T also showed that the median progression-free survival (PFS) in the combined treatment group tended to prolong, although the difference did not reach statistical significance^[19]^. These results suggest that immunity combined with other treatments may improve therapeutic efficacy by enhancing the immune response or altering the tumor microenvironment. The same results were obtained from our meta-analysis. Compared with immunotherapy alone, combined treatment allows patients to obtain longer overall survival, median survival time and better PSA50 decline rate. But this may not be surprising, as both drugs are approved based on their clinical benefits. This suggests that the combination of radioimmunotherapy may add clinical effects, but does not demonstrate synergies^[9]^. However, this result needs to be interpreted with caution. This is because the results are based on nine original studies. On the one hand, the number of studies and the study sample are very limited, which may not allow the results to be generalized on a large scale. On the other hand, the studies are of an earlier vintage, and management and treatment strategies for mCRPC patients are constantly updated, which inevitably leads to some heterogeneity in the treatment background of the included mCRPC patients. This may also be the source of the high heterogeneity of our findings (e.g., OS, PSA50).

However, the effects of combined immunotherapy are not consistent. In the study of combined treatment with radium-223 and pembrolizumab, although the PSA50 response rate was improved in the combined treatment group, no significant increase in immune cell infiltration or significant improvement in clinical efficacy was observed^[15]^. This may be related to the limited immune regulation effect of radium-223, or the inappropriate timing and dose selection of combined treatment. In addition, in the study of sipuleucel-T combined with radiotherapy, although there was a trend of prolongation in PFS in the combined treatment group, no significant advantage was shown in terms of immune activation parameters (such as upregulation of CD54+ cells)^[19]^. This suggests that the effectiveness of immune-combination therapies may be influenced by a number of factors, including the baseline characteristics of the patient, the immunogenicity of the tumor, and the specific regimen of the combination therapy. These questions have not yet been clearly answered, and more large-scale multicenter studies are needed to conduct in-depth research in terms of general patient characteristics and specific immunocombination therapy regimens, so as to lay a solid foundation for guiding the practice of clinical management of mCRPC patients.

It is worth noting that some studies have found that patients’ previous treatment experiences may affect the effectiveness of immune combination therapy. For example, in the study of sipuleucel-T combined with ipilimumab, patients who had previously received prostate radiotherapy had lower proportions of CTLA-4-positive T cells in peripheral blood before treatment, and these patients were more likely to benefit from combination therapy^[17]^. This suggests that previous local treatments may create more favorable conditions for subsequent immune combination therapy by changing the immune microenvironment. In addition, some studies have found that DNA repair defects (DRD) status may be related to the efficacy of immunotherapy, but this result is not consistent in different studies^[16]^. More multicenter, high-quality studies are much needed due to the disparate findings. Predictive markers of efficacy of different immune combination therapies as well as oncological outcomes need to be further explored for more precise treatment.

As the first meta-analysis comparing the combination of immunotherapy and immunotherapy alone, our study also has some shortcomings, as follows: First, the limited number of included studies and study samples, as well as the earlier year of the study and the potential for greater variability in terms of patient treatment strategies, lead to the need for further validation of the reliability and clinical significance of our findings. Second, the current limitations in the number of studies prevented us from splitting the combination therapies in detail, perhaps a source of the high heterogeneity of results in some studies. Therefore, please interpret our findings with caution when it comes to exactly what therapeutic regimen to combine. Third, due to the limitations of the original research data, we cannot conduct statistical analysis of outcomes such as the objective tumor remission rate. We hope that more research will pay attention to this topic in the future, and we will continue to update it.

## Conclusion

Overall, immunotherapy combined with other treatments improved oncological outcomes in patients with metastatic castration-resistant prostate cancer without causing additional adverse effects. However, a more detailed split of combination treatment should be further carried out.

## Data Availability

None.

## Research registration unique identifying number (UIN)

PROSPERO registration ID: CRD420251054916 (https://www.crd.york.ac.uk/prospero/display_record.php?ID=CRD420251054916).

## References

[1] Dong Z Xue K Verma A. Photothermal therapy: a novel potential treatment for prostate cancer. Biomater Sci 2024;12:2480–503. 38592730 10.1039/d4bm00057a

[2] Takahashi S. Current trend of CAR-T cell therapy for metastatic castration-resistant prostate cancer. Gan To Kagaku Ryoho 2023;50:1038–42. 38035830

[3] Calvo E Doger B Carles J. A first-in-human study of JNJ-70218902, a bispecific T-cell-redirecting antibody against TMEFF2 in metastatic castration-resistant prostate cancer. Oncologist 2025;30:oyae313. 39832129 10.1093/oncolo/oyae313 PMC11745015

[4] Ganguly K Metkari SM Biswas B Subedi R Madan T. Intra-tumoral delivery of 5ʹppp-dsRNA induces a robust antitumor response via RIG-I activation and Bcl-2 gene downregulation in a murine model of prostate cancer. Int Immunol 2024;37:109–29. 39387130 10.1093/intimm/dxae061

[5] Saeed MA Peng B Kim K. High-dimensional analyses reveal IL15 enhances activation of sipuleucel-T lymphocyte subsets and reverses immunoresistance. Cancer Immunol Res 2024;12:559–74. 38407894 10.1158/2326-6066.CIR-23-0652

[6] Lin G Elkashif A Saha C Coulter JA Dunne NJ McCarthy HO. Key considerations for a prostate cancer mRNA vaccine. Crit Rev Oncol Hematol 2025;208:104643. 39900315 10.1016/j.critrevonc.2025.104643

[7] Galsky MD Autio KA Cabanski CR. Clinical and translational results from PORTER, a multi-cohort phase 1 platform trial of combination immunotherapy in metastatic castration-resistant prostate cancer. Clin Cancer Res 2025;31:1463–75. 39964352 10.1158/1078-0432.CCR-24-3693 PMC11995007

[8] Slovin SF Higano CS Hamid O. Ipilimumab alone or in combination with radiotherapy in metastatic castration-resistant prostate cancer: results from an open-label, multicenter phase I/II study. Ann Oncol 2013;24:1813–21. 23535954 10.1093/annonc/mdt107 PMC3707423

[9] Marshall CH Fu W Wang H. Randomized phase II trial of sipuleucel-T with or without radium-223 in men with bone-metastatic castration-resistant prostate cancer. Clin Cancer Res 2021;27:1623–30. 33451978 10.1158/1078-0432.CCR-20-4476 PMC8121020

[10] Premier Science LUK Agha R Mathew G. Transparency In The reporting of Artificial INtelligence – the TITAN guideline. Prem J Sci 2025;10:100082.

[11] Page MJ McKenzie JE Bossuyt PM. The PRISMA 2020 statement: an updated guideline for reporting systematic reviews. J Clin Epidemiol 2021;134:178–89. 33789819 10.1016/j.jclinepi.2021.03.001

[12] Shea BJ Reeves BC Wells G. AMSTAR 2: a critical appraisal tool for systematic reviews that include randomised or non-randomised studies of healthcare interventions, or both. Bmj 2017;358:j4008. 28935701 10.1136/bmj.j4008 PMC5833365

[13] Wells G Shea B O’Connell D. The NewcastleOttawa Scale (NOS) for Assessing the Quality of Nonrandomised Studies in Meta-analyses. 2021. Ottawa Hospital Research Institute.

[14] Aggarwal R Starzinski S de Kouchkovsky I. Single-dose (177)Lu-PSMA-617 followed by maintenance pembrolizumab in patients with metastatic castration-resistant prostate cancer: an open-label, dose-expansion, phase 1 trial. Lancet Oncol 2023;24:1266–76. 37922930 10.1016/S1470-2045(23)00451-5 PMC10667020

[15] Choudhury AD Kwak L Cheung A. Randomized phase II study evaluating the addition of pembrolizumab to radium-223 in metastatic castration-resistant prostate cancer. Cancer Immunol Res 2024;12:704–18. 38552171 10.1158/2326-6066.CIR-22-0306 PMC11148544

[16] Pachynski RK Morishima C Szmulewitz R. IL-7 expands lymphocyte populations and enhances immune responses to sipuleucel-T in patients with metastatic castration-resistant prostate cancer (mCRPC). J Immunother Cancer 2021;9:e002903. 34452927 10.1136/jitc-2021-002903 PMC8404457

[17] Shenderov E Boudadi K Fu W. Nivolumab plus ipilimumab, with or without enzalutamide, in AR-V7-expressing metastatic castration-resistant prostate cancer: a phase-2 nonrandomized clinical trial. Prostate 2021;81:326–38. 33636027 10.1002/pros.24110 PMC8018565

[18] Sinha M Zhang L Subudhi S. Pre-existing immune status associated with response to combination of sipuleucel-T and ipilimumab in patients with metastatic castration-resistant prostate cancer. J Immunother Cancer 2021;9:e002254. 33986125 10.1136/jitc-2020-002254 PMC8126308

[19] Twardowski P Wong JYC Pal SK. Randomized phase II trial of sipuleucel-T immunotherapy preceded by sensitizing radiation therapy and sipuleucel-T alone in patients with metastatic castrate resistant prostate cancer. Cancer Treat Res Commun 2019;19:100116. 30682445 10.1016/j.ctarc.2018.100116

[20] Wargowski E Johnson LE Eickhoff JC. Prime-boost vaccination targeting prostatic acid phosphatase (PAP) in patients with metastatic castration-resistant prostate cancer (mCRPC) using sipuleucel-T and a DNA vaccine. J Immunother Cancer 2018;6:21. 29534736 10.1186/s40425-018-0333-y PMC5850960

[21] Nussbaum N George DJ Abernethy AP. Patient experience in the treatment of metastatic castration-resistant prostate cancer: state of the science. Prostate Cancer Prostatic Dis 2016;19:111–21. 26832363 10.1038/pcan.2015.42 PMC4868871

[22] Yan K Balijepalli C Gullapalli L Joshy J Kotum S Druyts E. Efficacy and safety of interventions for metastatic castration resistant prostate cancer (mCRPC) patients progressing on androgen receptor-axis-targeted (ARAT) therapy: a systematic literature review. Curr Med Res Opin 2024;40:1741–52. 39166959 10.1080/03007995.2024.2395435

[23] Heidegger I Necchi A Pircher A. A systematic review of the emerging role of immune checkpoint inhibitors in metastatic castration-resistant prostate cancer: will combination strategies improve efficacy? Eur Urol Oncol 2021;4:745–54. 33243663 10.1016/j.euo.2020.10.010

[24] Groves-Kirkby N. Prostate cancer. Immunotherapy and combined chemotherapy for castration-resistant and metastatic disease. Nat Rev Urol 2010;7:472. 10.1038/nrurol.2010.124 20839381

